# Multispectral Differential Reconstruction Strategy for Bioluminescence Tomography

**DOI:** 10.3389/fonc.2022.768137

**Published:** 2022-02-18

**Authors:** Yanqiu Liu, Mengxiang Chu, Hongbo Guo, Xiangong Hu, Jingjing Yu, Xuelei He, Huangjian Yi, Xiaowei He

**Affiliations:** ^1^ The Xi’an Key Laboratory of Radiomics and Intelligent Perception, Xi’an, China; ^2^ School of Information Sciences and Technology, Northwest University, Xi’an, China; ^3^ Network and Data Center, Northwest University, Xi’an, China; ^4^ School of Physics and Information Technology, Shaanxi Normal University, Xi’an, China

**Keywords:** bioluminescence tomography, multispectral, eliminate errors, spectral differential, source reconstruction

## Abstract

Bioluminescence tomography (BLT) is a promising *in vivo* molecular imaging tool that allows non-invasive monitoring of physiological and pathological processes at the cellular and molecular levels. However, the accuracy of the BLT reconstruction is significantly affected by the forward modeling errors in the simplified photon propagation model, the measurement noise in data acquisition, and the inherent ill-posedness of the inverse problem. In this paper, we present a new multispectral differential strategy (MDS) on the basis of analyzing the errors generated from the simplification from radiative transfer equation (RTE) to diffusion approximation and data acquisition of the imaging system. Through rigorous theoretical analysis, we learn that spectral differential not only can eliminate the errors caused by the approximation of RTE and imaging system measurement noise but also can further increase the constraint condition and decrease the condition number of system matrix for reconstruction compared with traditional multispectral (TM) reconstruction strategy. In forward simulations, energy differences and cosine similarity of the measured surface light energy calculated by Monte Carlo (MC) and diffusion equation (DE) showed that MDS can reduce the systematic errors in the process of light transmission. In addition, in inverse simulations and *in vivo* experiments, the results demonstrated that MDS was able to alleviate the ill-posedness of the inverse problem of BLT. Thus, the MDS method had superior location accuracy, morphology recovery capability, and image contrast capability in the source reconstruction as compared with the TM method and spectral derivative (SD) method. *In vivo* experiments verified the practicability and effectiveness of the proposed method.

## 1 Introduction

Bioluminescence imaging (BLI), applied in preclinical molecular imaging of small animals, has attracted widespread attention in biological and medical research (1). The non-radiation imaging method has the advantages of fast feedback, high sensitivity, high temporal resolution, and high specificity, which is often used in molecular, cellular, and gene expression imaging studies to facilitate drug development disease research and therapeutic interventions (2–4). However, BLI can only detect the two-dimensional body surface information, which is not sufficient to quantify the activity of tumor cells in the bodies of living animals. Bioluminescence tomography (BLT) employs three-dimensional (3D) reconstruction of bioluminescent sources to more accurately locate and quantify tumors compared with BLI (5). The basic idea of BLT is to utilize a “forward” model of light propagation through the tissue to the skin surface, along with an “inversion” algorithm to reconstruct the underlying bioluminescence source distribution (6, 7). In the process, the accuracy of the BLT reconstruction is significantly affected by the forward modeling errors in the simplified photon propagation model, the measurement noise in data acquisition, and the inherent ill-posedness of the inverse problem.

In the propagation process of light from the internal source to the imaging subject surface, the interaction between light and biological tissue includes absorption, reflection, scattering, refraction, and transmission (8). The radiative transfer equation (RTE) is widely accepted as an accurate model for photon migration in a turbid medium. Due to the computationally intensive nature of RTE, the most typical approach is using the diffusion equation (DE) as the forward model to approximate RTE (9). DE is basically a special case of the first-order spherical harmonics approximation to RTE, and it fails to produce accurate estimates for light propagation in the proximity of the source and boundaries, which leads to big errors in the reconstructed images. In several high-order approximate models of RTE, such as *S_N_, P_N_*, and *SP_N_*, the approximation is shown to have improved accuracy than DE (10). Although the *SP_N_* approximation leads to a lower computational load than either the *S_N_* or *P_N_* approximation, the number of unknowns to be solved is still several times larger than the DE’s. The higher-order approximation is used throughout the entire domain, bringing a higher computational load. In addition, hybrid models were studied to improve the modeling accuracy of photon transmission in biological tissues. Hybrid models based on radiance were proposed to solve the special problems of the non-scattering regions (11–13), hybrid Monte Carlo (MC)–diffusion method (14), hybrid RTE–diffusion approximation method (15), and hybrid DE– *SP_N_* method (16), which have been studied to solve the problem of light transmission in non-highly scattering regions and areas close to the source. However, transmission errors of various models due to RTE approximation are still inevitable.

For the propagation of light from the imaging subject surface to the optical detector, in the early research stage, optical signals were measured through contact configuration based on optical fiber guides for photon collection. However, such implementations led to insufficient spatial sampling and field of view, and poorer resolution and signal-to-noise ratio (17). Therefore, at present, all BLI systems are based on a non-contact configuration (7), which makes data collection more flexible but also has some disadvantages and limitations. It has been demonstrated that a change in position of the imaging subject can result in a differently measured signal, and due to the impact of charge-coupled device (CCD) noise and environmental background noise, the measurement accuracy of the optical signal is affected. To overcome measurement noise, a free-space model was used to describe the propagation of light from the surface of the imaging subject to the CCD. The contribution of each point on the surface of the imaging subject to each pixel in the CCD was described by a mapping matrix, then taking CCD data to be mapped back on the surface of the imaging subject by inverting this relationship, and determining true surface fluence values, which were independent of the position of the imaging subject (18). In addition, severa methods were adopted to suppress the noise of CCD, such as the typical median filter and the contourlet transform based on an efficient two-dimensional multiscale and directional filter bank (19). Besides, a novel iterative filtering method based on a “detection-corrosion” strategy was studied for degraded neutron image denoising (20), and a novel Laplacian of Gaussian (LoG) filter combined with the median filter was proposed to remove the gamma white spots (21). Further, graph convolutional networks and dictionary learning techniques for hyperspectral can also be used to overcome measurement noise and improve the accuracy of acquired spectral images (22–24). Moreover, the importance of the domain geometry and imaging subject position on the measured bioluminescence fluence was studied, and an image reconstruction algorithm based on the spectral derivative (SD) of the measured spectral data was proposed to overcome the measurement noise from the surface of the imaging subject to the CCD (7). For the spectral-derivative method, the ratio of the BLIs at adjacent wavelengths was used as input data for the source reconstruction, as bioluminescence at similar wavelengths encounters a near-identical system response (25).

In addition, in practical biological applications, due to the scattering effect of light and the limitations of the detected surface photons, the inverse problem of BLT is limited by its ill-posedness, which significantly affects the accuracy of the BLT reconstruction (5, 26). Based on the fundamental feature of BLT, without the incorporation of effective *a priori* knowledge on the source distribution, there would be no hope to determine a unique solution (27). All possible information on the source distribution must be utilized to achieve the best possible reconstruction for BLT, and it is essential to combine regularization techniques to overcome the ill-posedness when trying to recover source distribution from noisy measurements. Most BLT regularization methods, such as Tikhonov regularization, try to stabilize the problem by achieving a trade-off between a loss term and an *L_2_*-norm regularization term. However, these methods usually produce smooth solutions (28). In recent years, more studies focused on sparse reconstruction algorithms to alleviate the ill-posedness of BLT (29–31); they were mainly based on the *L_1_*-norm regularization, such as the homotopy method (29), Bayesian sparse based method (31), and LASSO method (30). These algorithms can overcome the over-smooth effects of *L_2_*-norm methods and encourage more sparse and stable reconstructed results, which improved the localization accuracy of the tumor. Further, to pursue more sparse solutions than *L_1_* regularizer, *L_p_*-norm regularization algorithms with (0 < p < 1) were studied, such as weighted interior-point algorithm (32) and majorization–minimization algorithm (33). Furthermore, in order to reduce the ill-posedness of BLT, the multispectral strategy has attracted remarkable attention. Spectrally resolved measurements consider the wide emission-spectrum characteristic of bioluminescent reporters and the diversity of tissue optical properties for different spectral bands, which can increase the amount of known boundary data. So a consensus that multispectral strategy can enhance the uniqueness and stability of BLT solution has been achieved (34). These studies provide ideas and a basis for our research.

In order to alleviate the influence of optical transmission errors, measurement noise, and ill-posedness on BLT reconstruction accuracy, based on spectral differential theory and combined with multispectral strategy, a new multispectral differential strategy (MDS) is presented in this work. MDS assumes that in the same optical transmission approximation model, light encounters a near-identical system response at a similar wavelength, and the errors of the adjacent spectral are similar. Thus, taking DE as an example, after analyzing the errors that resulted from diffusion approximation and data acquisition of the imaging system, MDS is expected to eliminate the systematic errors of the optical transmission model and imaging system. Meanwhile, compared with the traditional multispectral (TM) reconstruction strategy, MDS can increase the constraint condition of the system matrix and alleviate the ill-posedness of the reconstruction problem. To solve the source reconstruction problem, *L_p_* regularization reconstruction model will be established, and a non-convex sparse regularization algorithm (nCSRA) will be utilized. To verify the feasibility and applicability of MDS, numerical simulations and *in vivo* experiments will be conducted with the TM method and SD method as comparisons.

The paper is organized as follows. In *Section 2*, we will analyze the errors that resulted from DE approximation and data acquisition of the imaging system, and then the MDS method and nCSRA framework will be provided. *Section 3* will present some relative experimental designs, the results of numerical simulations, and *in vivo* imaging experiments. Finally, some discussions and conclusions will be made for this paper in *Section 4*.

## 2 Methods

### 2.1 Error Analysis of Optical Signal Acquisition Process

In BLI, for the measurement of emitted light due to an internal light source, the transmission process of light and the acquisition technology of optical signal are two key factors that need to be considered, which significantly affect the accuracy of the BLT source reconstruction. Next, the optical transmission errors caused by RTE approximation, and the measurement noise of photon fluence rate on imaging subject surface caused by CCD noise, will be analyzed in detail.

#### 2.1.1 Errors Caused by the Simplified Photon Propagation Model

The RTE treats photons as particles that undergo elastic collisions until they are absorbed or leave the domain, ignoring the wave nature of light. After removal of the influence of time, steady-state RTE is of the form


(1) $$\hat{s}\cdot \nabla \phi (r,\hat{s})+\mu_{tr}\phi (r,\hat{s})-\mu_{s}\int_{4\pi }\Theta (\hat{s}\cdot {\hat{s}}^{\prime })\phi (r,{\hat{s}}^{\prime })d\Omega^{\prime }-q(r,\hat{s})=0$$

where Φ(*r*,ŝ) is the radiance, *q*(*r*,ŝ) is the source inside Ω, and the kernel $\Theta (\hat{s}\cdot {\hat{s}}^{\prime })$ is the scattering phase function that describes the probability that a photon with an initial direction ${\hat{s}}^{\prime }$ will have a direction $\hat{s}$ after a scattering event (9). *μ_s_* is the scattering coefficient, and *μ_tr_* = *μ_a_* + *μ_s_* is the transport coefficient.

Due to the computationally intensive nature of RTE, the typical approach is using DE as the forward model to approximate RTE. In order to get the DE, which is the first-order approximate model of RTE, take the radiance spherical harmonic expansion as (35)


(2) $$\phi (r,\hat{s})=\sum_{\infty }^{l}\sum_{l}^{m=-1}{\left(\frac{2l+1}{4\pi }\right)}^{\frac{1}{2}}\psi_{l,m}(r)Y_{l,m}(\hat{s})$$

where the normalization factor ((2*l* + 1)/4π)^1/2^ is introduced for convenience. We used the Associated Legendre polynomials, and we expand $\phi (r,\hat{s})$ to first-order (8)


(3) $$\phi_{1}(r,\hat{s})=\frac{1}{4\pi }\Phi (r)+\frac{3}{4\pi }J(r)\cdot \hat{s}$$

We have


(4) $$\left\{ \begin{array}{c} \phi (r,\hat{s})=\phi_{1}(r,\hat{s})+\Delta \phi \\ \phi (r,{\hat{s}}^{\prime })=\phi_{1}(r,{\hat{s}}^{\prime })+\Delta \phi^{\prime } \end{array}\right.$$

We operate on Eq. (1) by $\int_{4\pi }(\cdot )d\Omega$ and $\int_{4\pi }\hat{s}(\cdot )d\Omega$ and make use of the relations in them to arrive at (8)


(5) $$\begin{array}{l} -\nabla \cdot [D\nabla \Phi (r)]-3\nabla D\int_{4\pi }\hat{s}(\hat{s}\cdot \nabla (\Delta \phi ))d\Omega +3\nabla D\mu_{s}\int_{4\pi }\int_{4\pi }\hat{s}[\Theta \left(\hat{s}\cdot {\hat{s}}^{'}\right)(\Delta \phi^{\prime })d\Omega^{\prime }]d\Omega \\ +\mu_{a}Ф(r)-\mu_{s}\int_{4\pi }[\int_{4\pi }\Theta (\hat{s}\cdot {\hat{s}}^{\prime })\Delta \phi^{\prime }d\Omega^{\prime }]d\Omega -q(r)=0 \end{array}$$

where photon density $\Phi (r)=\int_{4\pi }\phi (r,\hat{s})d\Omega$ , and $D=\frac{1}{3(\mu_{a}+{\mu_{s}}^{\prime })}$ is diffusion coefficient. In the study of BLT, the light source can be regarded as isotropic, $q(r)=4\pi q(r,\hat{s})$ .

The well-known steady-state DE is


(6) $$-\nabla \cdot [D\nabla \Phi (r)]+\mu_{a}\Phi (r)-q(r)=0$$

The finite element method (FEM) is chosen to solve the DE, and the FE approximation of the DE is of the form


(7) $$AS=\Phi^{m}$$

where *A* represents the system matrix whose construction is influenced by the DE approximation, *S* is the power density of the light source, and Φ*^m^* is the measurable photon fluence rate on boundary nodes.

In order to analyze the error of constructing *A*, the approximation from Eq. (5) to steady-state DE Eq. (6) due to radiance with only first-order spherical harmonic expansion, and the optical transmission error terms caused by DE approximation can be expressed as


(8) $$E_{A}=-3D\nabla \int_{4\pi }\hat{s}(\hat{s}\cdot \nabla (\Delta \phi ))d\Omega +3D\mu_{s}\nabla \int_{4\pi }\int_{4\pi }\hat{s}[\Theta \left(\hat{s}\cdot {\hat{s}}^{\prime }\right)(\Delta {Ф}^{\prime })d\Omega^{\prime }]d\Omega -\mu_{s}\int_{4\pi }[\int_{4\pi }\Theta \left(\hat{s}\cdot {\hat{s}}^{\prime }\right)\Delta \phi^{\prime }d\Omega^{\prime }]d\Omega$$

In order to reduce the error caused by diffusion approximation, the spectral differential is adopted. In Eq. (8), *μ_s_* and *D* are dependent on the given wavelength, for wavelength *λ*, and Eq. (8) can be expressed as


(9) $$E_{A\lambda }=-3D_{\lambda }\nabla \int_{4\pi }\hat{s}(\hat{s}\cdot \nabla (\Delta \phi ))d\Omega +3D_{\lambda }\mu_{s\lambda }\nabla \int_{4\pi }\int_{4\pi }\hat{s}[\Theta \left(\hat{s}\cdot {\hat{s}}^{\prime }\right)(\Delta \phi^{\prime })d\Omega^{\prime }]d\Omega -\mu_{s\lambda }\int_{4\pi }[\int_{4\pi }\Theta \left(\hat{s}\cdot {\hat{s}}^{\prime }\right)\Delta \phi^{\prime }d\Omega^{\prime }]d\Omega$$

Assuming that there are two wavelengths, *λ_j_* and *λ_k_*, the difference between the spectra is used to operate the transmission error corresponding to the two wavelengths,


(10) $$\begin{array}{l} E_{Adiffe}=E_{A\lambda_{j}}-E_{A\lambda_{k}}=3(-D_{\lambda_{j}}+D_{\lambda_{k}})\nabla \int_{4\pi }\hat{s}(\hat{s}\cdot \nabla (\Delta \phi ))d\Omega \\ +3(D_{\lambda_{j}}\mu_{s\lambda_{j}}-D_{\lambda_{k}}\mu_{s\lambda_{k}})\nabla \int_{4\pi }\int_{4\pi }\hat{s}[\Theta \left(\hat{s}\cdot {\hat{s}}^{\prime }\right)(\Delta {Ф}^{\prime })d\Omega^{\prime }]d\Omega -(\mu_{s\lambda_{j}}-\mu_{s\lambda_{k}})\int_{4\pi }[\int_{4\pi }\Theta \left(\hat{s}\cdot {\hat{s}}^{\prime }\right)\Delta {Ф}^{\prime }d\Omega^{\prime }]d\Omega \end{array}$$

In general, for similar emission wavelengths in biological tissues, the optical parameters are similar, that is $(D_{\lambda_{j}}-D_{\lambda_{k}})\ll D_{\lambda_{j}(k)},(\mu_{s_{\lambda j}}-\mu_{s_{\lambda k}})\ll \mu_{s_{\lambda_{j}(k)}},\text{and }(D_{\lambda_{j}}\mu_{s_{\lambda j}}-D_{\lambda_{k}}\mu_{s_{\lambda k}})\ll D_{\lambda_{j}(k)}\mu_{s_{\lambda j(k)}},$ so


(11) $$E_{A_{diffe}}\ll E_{A_{\lambda_{j}}}or\;E_{A_{\lambda_{k}}}$$

#### 2.1.2 Measurement Noise

For the photon fluence rate Φ on the imaging subject surface obtained by CCD, there are inevitable errors caused by CCD noise. The main types of noise are as follows: discrete and incomplete sampling errors of CCD (*s*), photon noise (*p*), readout noise (*r*), and dark current noise (*d*) (36).

The measured signal on the imaging subject surface can be expressed as


(12) Φ=M+s+Ot

where *M* is the actual optical signal and the rest of the items are measurement noise. *i* relates to the Gaussian width of the imaging light spot, and different emission wavelengths produce different width light spots, so *s* depends on the wavelength of emitting light. *Ot*=*p+r+d* caused by the CCD itself, which is wavelength independent.

For the test data at the wavelength *λ*, the measurement signal is expressed as


(13) $${Ф}_{\lambda }=M_{\lambda }+\underset{E_{{Ф}_{\lambda }}}{\underbrace{s_{\lambda }+Ot}}$$

where $E_{\Phi_{\lambda }}$ is the measurement noise.

The difference between the corresponding measurement noise of the two spectra with the wavelengths of *λ_j_* and *λ_k_* is calculated as


(14) $$E_{\Phi_{diffe}}=E_{\Phi_{\lambda_{j}}}-E_{\Phi_{\lambda_{k}}}=s_{\lambda_{j}}-s_{\lambda_{k}}$$

Only the difference of discrete and incomplete sampling errors of CCD needs to be considered, and for similar emission wavelengths, there is $(s_{\lambda_{j}}-s_{\lambda_{k}})\ll s_{\lambda_{j}(k)}+Ot$ ; that is,


(15) $$E_{\Phi_{diffe}}\ll E_{\Phi_{\lambda_{j}}}or\;E_{\Phi_{\lambda_{k}}}$$

Eq. (11) and Eq. (15) indicate that introducing the difference of data between each measured wavelength can reduce optical transmission system errors and measurement noise.

### 2.2 Multispectral Differential Strategy

The factors affecting the source reconstruction results have been analyzed, and the data difference between each measured wavelength can be used to reduce errors. Next, based on the DE model, the multispectral differential is further applied in the source reconstruction.

The imaging problem is known to be non-unique, and *A* in Eq. (7) is ill-posed. In order to reduce its ill-posedness, the multispectral hybrid method has been proposed. For example, assuming that there are measurements of four wavelengths, these system matrices are combined to obtain the following equations:


(16) $$\left[\begin{array}{c} \eta_{1}A_{\lambda 1}\\ \eta_{2}A_{\lambda 2}\\ \eta_{3}A_{\lambda 3}\\ \eta_{4}A_{\lambda 4} \end{array}\right]\cdot S=\left[\begin{array}{c} {Ф}_{\lambda 1}^{m}\\ {Ф}_{\lambda 2}^{m}\\ {Ф}_{\lambda 3}^{m}\\ {Ф}_{\lambda 4}^{m} \end{array}\right]$$

(17) $$A_{multi}S=\Phi_{multi}^{m}$$

where *A_λn_* is the system matrix at a given wavelength *λn*, and *A_multi_* is their combinatorial system matrix. $\Phi_{\lambda n}^{m}$ is the measurable photon fluence rate (on the surface) at the same wavelength, $\Phi_{multi}^{m}$ is the combinatorial photon fluence rate, and *η_n_* is the ratio of each spectral energy to the total. In this way, the combinatorial system matrix is used to partly solve the ill-posed problem.

Different from the multispectral hybrid method using data at each given wavelength, multispectral differential utilizes the difference of data between each measured wavelength. Eq. (16) can be transformed as


(18) $$\left[\begin{array}{c} \eta_{1}A_{\lambda 1}-\eta_{2}A_{\lambda 2}\\ \eta_{1}A_{\lambda 1}-\eta_{3}A_{\lambda 3}\\ \eta_{1}A_{\lambda 1}-\eta_{4}A_{\lambda 4}\\ \eta_{2}A_{\lambda 2}-\eta_{3}A_{\lambda 3}\\ \eta_{2}A_{\lambda 2}-\eta_{4}A_{\lambda 4}\\ \eta_{3}A_{\lambda 3}-\eta_{4}A_{\lambda 4} \end{array}\right]\cdot S=\left[\begin{array}{c} {Ф}_{\lambda 1}^{m}-{Ф}_{\lambda 2}^{m}\\ {Ф}_{\lambda 1}^{m}-{Ф}_{\lambda 3}^{m}\\ {Ф}_{\lambda 1}^{m}-{Ф}_{\lambda 4}^{m}\\ {Ф}_{\lambda 2}^{m}-{Ф}_{\lambda 3}^{m}\\ {Ф}_{\lambda 2}^{m}-{Ф}_{\lambda 4}^{m}\\ {Ф}_{\lambda 3}^{m}-{Ф}_{\lambda 4}^{m} \end{array}\right]$$

(19) $$A_{diffe}S=\Phi_{diffe}^{m}$$

where *A_diffe_* and $\Phi_{diffe}^{m}$ are a combination of differential system matrices and differential photon fluence rates, respectively. It can be seen by comparing Eq. (16) and Eq. (18) that the multispectral differential can increase the constraint condition for inverse reconstruction compared with the TM reconstruction strategy. Thus, it has reduced the system errors and measurement noise to alleviate the ill-posedness of the source reconstruction.

### 2.3 Non-Convex Sparse Regularization Algorithm Framework

Considering the sparse distribution of light sources in organisms and the serious shortage of surface measurement based on compressed sensing theory, *L_p_* regularization is adopted to transform the reconstruction model of Eq. (19) into the following minimization problem:


(20) $$\underset{S}{\min}\frac{1}{2}{\left\|A_{diffe}S-\Phi^{m}{\;}_{diffe}\right\|}_{2}^{2}+\tau {\left\|S\right\|}_{p}^{p},0<P<1$$

where ${\left\|S\right\|}_{p}={\left(\Sigma_{i=1}^{n}{\left|S_{i}\right|}^{p}\right)}^{1/p}$ represents the *L_p_* quasi-norm and τ > 0 is a regularization parameter. Because the *L_p_*-norm regularization is a non-convex and no-smooth optimization, an nCSRA (37) is utilized to solve this problem. It is converted to a weighted *L*_1_-norm regularization:


(21) $$S^{t+1}=\underset{S}{\min}\frac{1}{2}{\left\|A_{diffe}S-\Phi^{m}{\;}_{diffe}\right\|}_{2}^{2}+\sum_{n}^{i=1}\lambda_{1}\left|S_{i}\right|$$

where $\lambda_{i}=\frac{\zeta_{i}}{{\left|X_{i}^{t}\right|}^{1-p}},\zeta_{i}$ is the *i*th element of regularization parameter ζ.

Then Eq. (21) as *L*_1_-norm regularization problem can be solved by the iterative shrinkage-thresholding algorithm (ISTA) (38). ISTA is based on the shrinkage function: *shrink*(*a*, *z*) = *max*(*a* –*z*, 0)*sign(*z*). With a sufficiently small step size ξ, the analytical solution of Eq. (21) can be derived as


(22) $$\{\begin{array}{l} S_{i}^{t+1}=shrink({\left(S^{t}+2\xi A^{T}(Ф-AS)\right)}_{i},\xi \lambda_{i})\\ \xi <1/||A^{T}A|{|}_{2} \end{array}$$

## 3 Experiments and Results

In this section, the digital mouse simulations and *in vivo* experiments were designed to evaluate the performance of MDS in BLT reconstruction. All programs were run on a computer with an Intel^®^ Core™ i7-6700 CPU (3.40 GHz) and 16-GB RAM.

### 3.1 Numerical Simulation Setup

**Figure 1:**
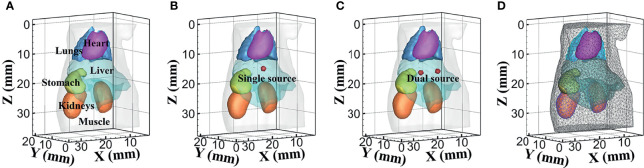
The numerical simulations setup. **(A)** The mouse model with six organs. **(B, C)** Model of single source and dual sources. **(D)** The mesh used for reconstruction.

Numerical forward simulations and inverse reconstructions both used a digital mouse model with a height of 35 mm, and only the torso section of the mouse was investigated, including the muscle, heart, liver, lungs, stomach, and kidneys, as shown in **Figure 1A**. Four wavelengths of 610, 630, 650, and 670 nm were used in simulations, and the specific optical parameters at each wavelength are listed in **Table 1**. The optical properties were calculated using the formula summarized in (39). In single-source simulations, a sphere with a radius of 1 mm was positioned at coordinates (18 mm, 8 mm, 14.8 mm), as shown in **Figure 1B**. In the dual-source simulations, two spheres with a radius of 1 mm positioned at coordinates (15 mm, 7 mm, 15.8 mm) and (22 mm, 7 mm, 15.8 mm) are shown in **Figure 1C**. In forward simulations, a discretized tetrahedral mesh with 20, 263 nodes and 106, 656 tetrahedral elements was used for the single-source simulations, while a mesh in dual-source simulations had 19, 890 nodes and 104, 619 tetrahedral elements. In the reconstruction, a mesh with 10,139 nodes and 51,841 tetrahedral elements was used as shown in **Figure 1D**.

**Table 1:** Optical parameters of the mouse tissues at different wavelengths.

Tissue	*610 nm*		*630 nm*		*650 nm*		*670 nm*	
	*μ_a_* (mm^−1^)	*μ_s_* (mm^−1^)	*μ_a_* (mm^−1^)	*μ_s_* (mm^−1^)	*μ_a_* (mm^−1^)	*μ_s_* (mm^−1^)	*μ_a_* (mm^−1^)	*μ_s_* (mm^−1^)
**Muscle**	0.2971	5.5902	0.1605	5.1041	0.1164	4.6735	0.0870	4.2907
**Heart**	0.2015	7.3484	0.1085	7.0171	0.0786	6.7104	0.0588	6.4258
**Stomach**	0.0384	19.6728	0.0207	19.0667	0.0150	18.4973	0.0114	17.9615
**Liver**	1.2086	7.4826	0.6505	7.2334	0.4708	6.9999	0.3815	6.7807
**Kidneys**	0.2258	18.5421	0.1216	17.6605	0.0881	16.8465	0.0660	16.0929
**Lungs**	0.6687	38.0785	0.3622	37.4330	0.2630	36.8181	0.1964	36.2314

### 3.2 *In Vivo* Experiment Setup

To further assess the performance of MDS, an *in vivo* BLT experiment was performed. The animal experiment was conducted under the approval of the Animal Ethics Committee of the Northwest University of China (No. NWU-AWC-20210901M). A female BALB/c nude mouse (4–5 weeks old) was used to establish a source implanted mouse model. After the mouse was anesthetized with pentobarbital (50 mg/kg, 0.1 ml, IP injection), a transversal incision was made in the abdomen. A plastic tube filled with about 20 μl of luminescent solution was implanted in the abdomen of the nude mouse. The luminescent solution was extracted from a luminescent light stick. After the incision was sutured, the mouse was taped on the rotation stage for imaging. The entire surgical procedure lasted approximately 10 min.

During the data collection, bioluminescent images and CT data were collected by the BLT/Micro-CT dual-mode system developed by our laboratory (40). The optical images were captured by the EMCCD camera (iXon Ultra, Andor, Northern Ireland, UK) with bandpass filters measuring 610, 630, 650, and 670 nm. The cooling temperature of the EMCCD camera was 80°C, which could effectively reduce the thermal noise. The software “Solis Acquisition and Analysis Software” was used for data collection. Photographic images and bioluminescent light distribution images with exposure times of each wavelength were set to 0.75 and 30 s, respectively. During the luminescence imaging, the system was enclosed within a light-tight environment to avoid outside light. After the optical measurement data were obtained, the mouse was kept motionless and scanned by a micro-focus cone beam X-ray source (L9181-02, Hamamatsu Photonics, Hamamatsu Japan). In X-ray scanning, the X-ray source voltage and power were set as 90 kV and 27 W, respectively. With the use of an X-ray flat-panel detector (C7942CA-22, Hamamatsu Photonics, Hamamatsu Japan) for high-resolution CT imaging, a total of 600 X-ray projections were obtained with an interval of 0.6°, and each projection had an integrating time of 0.5 s; then the 3D anatomical structures were segmented from the CT data.

### 3.3 Quantitative Evaluation Index

Firstly, energy difference and cosine similarity were measured as quantitative evaluation indexes in forward simulations to analyze the model errors caused by DE. The surface bioluminescence distributions obtained by MC eXtreme (MCX) (41) are used as the standard for comparison. The energy difference in the measured data that were compared are calculated as follows:


(23) $$difference=\{\begin{array}{ll} \left|\Phi_{MC_{\lambda_{j(k)}}}-\Phi_{DE_{\lambda_{j(k)}}}\right| & Specific\;spectrum\\ \left|\left|\Phi_{MC_{\lambda_{j}}}-\Phi_{MC_{\lambda_{k}}}\right|-\left|\Phi_{DE_{\lambda_{j}}}-\Phi_{DE_{\lambda_{k}}}\right|\right| & Spectral\;differential \end{array}$$

It can be used to calculate the energy difference of surface light distributions obtained by MC and DE at the same wavelength, and the energy difference of differential data between each measured wavelength, which are obtained by MC and DE.

Cosine similarity is also used to evaluate the differences in surface light distributions:


(24) $$Simalarity=\cos(\theta )=\frac{A\cdot B}{\left\|A\right\|\left\|B\right\|}=\frac{\Sigma_{l=1}^{n}\;A_{l}\times B_{l}}{\sqrt{\Sigma_{l=1}^{n}{\left(A_{l}\right)}^{2}}\times \sqrt{\Sigma_{l=1}^{n}{\left(B_{l}\right)}^{2}}}$$

where *A_l_* and *B_l_* represent the components of the vectors *A* and *B*, which are compared, respectively. The closer the cosine gets to 1, the closer the two vectors are.

Besides, the condition number is a measure of the sensitivity of the solution to the linear system *AS*= Φ*^m^* to the error or uncertainty in Φ*^m^*. To verify whether the MDS can further increase the constraint condition and decrease the condition number of the system matrix for reconstruction, we calculate the condition number corresponding to the combinatorial system matrix *A* of three multispectral strategies.


(25) $$cond(A)=\left\|A\right\|\cdot \left\|A^{-1}\right\|$$

A problem with a lower condition number is said to be well-posed, and vice versa.

To quantitatively analyze the performance of MDS in the reconstruction, several common indicators are used, such as location error (LE), Dice coefficient, contrast-to-noise ratio (CNR), and Time.

LE represents the location deflection between the reconstructed light source and the actual light source:


(26) $$LE=\sqrt{{\left(x-x_{0}\right)}^{2}+{\left(y-y_{0}\right)}^{2}+{\left(z-z_{0}\right)}^{2}}$$

where (*x*, *y*, *z*) and (*x*_0_, *y*_0_, *z*_0_) are the coordinates of reconstruction energy weighted center point and the real source center, respectively.

Dice coefficient is used to evaluate shape recovery, which denotes similarity of the reconstructed sources regions *X* and the actual sources regions *Y*:


(27) $$Dice=\frac{2\left|X\cap Y\right|}{\left|X\right|+\left|Y\right|}$$

CNR is used to evaluate image contrast, which can be calculated, as follows:


(28) $$CNR=\frac{\left|\mu_{ROI}-\mu_{BCK}\right|}{{\left(w_{ROI}\sigma_{ROI}^{2}+w_{BCK}\sigma_{BCK}^{2}\right)}^{1/2}}$$

where the subscripts *ROI* and *BCK* denote the target and background regions of the imaged object, respectively; and *μ*, *w*, and *σ* represent the mean intensity value, weighting factor, and variance, respectively.

### 3.4 Numerical Simulations

#### 3.4.1 Forward Simulations

At first, a group of forward simulations was taken to analyze the errors involved in the whole optical transmission process and to demonstrate that the MDS can eliminate the errors to a certain extent.

**Figure 2:**
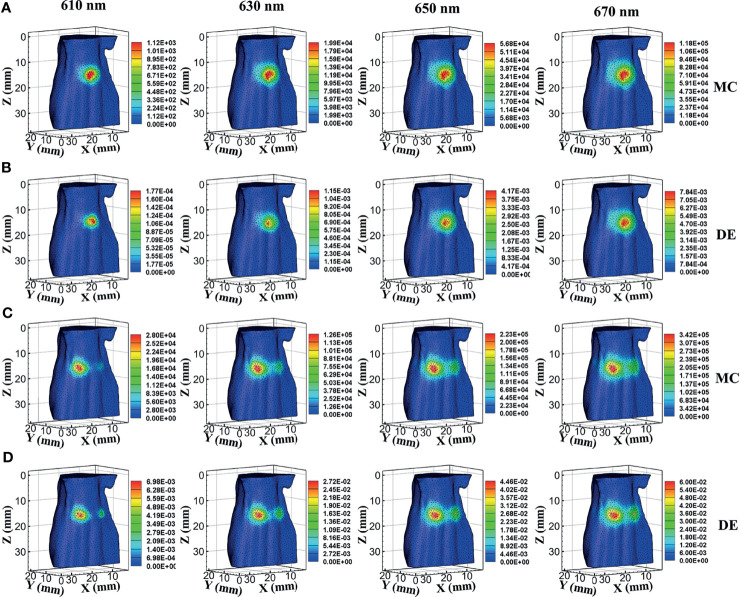
Results of forward simulations. **(A, B)** The bioluminescence distributions on the surface in single-source simulations by MC and DE methods at wavelengths of 610, 630, 650, and 670 nm. **(C, D)** The surface distributions in dual-source models by MC and DE methods at wavelengths of 610, 630, 650, and 670 nm. MC, Monte Carlo; DE, diffusion equation.

In a single-source case, the surface bioluminescence distributions at wavelengths of 610, 630, 650, and 670 nm generated with the MC method are shown in **Figure 2A**. For comparison, the surface bioluminescence distributions calculated by the DE model at different wavelengths are shown in **Figure 2B**. Similarly, **Figures 2C, D** show the surface bioluminescence distributions for dual-source cases calculated by MC and DE, respectively. In **Figures 2A–D**, for both MC and DE, the longer the wavelength, the more intense the surface light distribution, since the longer wavelength results in reduced tissue scattering effect and enhanced light penetration. By comparing the surface light distributions of MC and DE at the same wavelength in **Figures 2A, B**, we also find that the differences are obvious at 610- and 630-nm wavelengths, while they are relatively small at 650- and 670-nm wavelengths. Similar results can be seen in **Figures 2C, D**. This is because the liver is a tissue with high absorption characteristics at 610 and 630 nm, and the DE model in this case is not a proper choice, which led to a large error.

**Figure 3:**
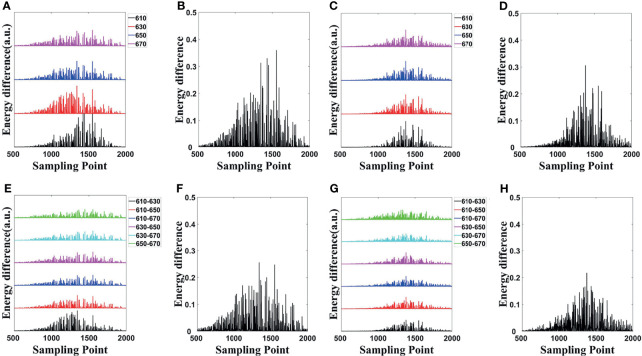
Energy differences of forward simulations. **(A, C)** The energy differences of MC and DE at the same wavelength in the single-source model and dual-source model. **(B, D)** Average of the corresponding four groups energy differences in **(A, C)**. **(E, G)** The energy differences after differential in single-source model and dual-source model. **(F, H)** Average of the corresponding six groups of energy differences in **(E, G)**. MC, Monte Carlo; DE, diffusion equation.

The energy differences of MC and DE at each wavelength in single- and dual-source models are shown in **Figures 3A, C**, respectively; and the average energy differences are shown in **Figures 3B, D**, respectively. Light energy for all the surface nodes was calculated. For ease of analysis, 1,500 of these nodes (501 to 2000) with obvious and concentrated energy differences were taken as the sampling points. Obviously, shorter wavelengths correspond to larger energy differences, and with the increase of wavelength, the energy difference tends to decrease. To demonstrate the effect of MDS on energy difference elimination, the differences of data between each measured wavelength were utilized. According to Eq. (23), any combination of two wavelengths was used; the six groups of energy differences after differential of single-source model and dual-source model are shown in **Figures 3E, G**, respectively; and the averages of the corresponding six groups energy differences are shown in **Figures 3F, H**. Compared with those in **Figure 3A**, the energy differences in **Figure 3E** decrease on the whole. As shown in **Figure 3F**, the average energy difference after the differential process is obviously less than that in **Figure 3B**. For the double-source model, the energy differences in **Figure 3G** decreased as compared with **Figure 3C**, and the average energy difference after differential in **Figure 3H** is obviously less than that in **Figure 3D** as well.

The cosine similarity of surface light energy obtained by MC and DE at the same wavelength was calculated, as shown in **Table 2**, which comes to the same conclusion as the energy differences in **Figures 3A, C**; i.e., a smaller energy difference corresponds to a cosine similarity closer to 1. The cosine similarities were calculated by the difference of data between each measured wavelength obtained by MC and DE, as also shown in **Table 2**. Compared with the cosine similarity of the specific spectrum, the cosine similarity value of spectral differential is closer to 1; Mean ± SD also indicates that the errors of spectral differential are smaller and the results are more stable.

**Table 2:** The cosine similarity of surface light energy obtained by MC and DE at the same wavelength and the cosine similarity were calculated by the difference of data between each measured wavelength obtained by MC and DE.

Single-source model				Dual-source model			
Specific spectrum	Cosine similarity	Spectral differential	Cosine similarity	Specific spectrum	Cosine similarity	Spectral differential	Cosine similarity
**610**	0.9270	**610–630**	0.9730	**610**	0.9545	**610–630**	0.9791
**630**	0.9789	**610–650**	0.9891	**630**	0.9758	**610–650**	0.9842
**650**	0.9889	**610–670**	0.9898	**650**	0.9824	**610–670**	0.9874
**670**	0.9896	**630–650**	0.9887	**670**	0.9863	**630–650**	0.9869
		**630–670**	0.9894			**630–670**	0.9893
		**650–670**	0.9899			**650–670**	0.9893
**Mean** ± **SD**	0.9711 ± 0.0298	**Mean** ± **SD**	0.9867 ± 0.0067	**Mean ± SD**	0.9747 ± 0.0142	**Mean ± SD**	0.9860 ± 0.0039

MC, Monte Carlo; DE, diffusion equation.

A quantitative comparison of the energy differences and cosine similarity shows that MDS can reduce the systematic error in forward simulations.

#### 3.4.2 Inverse Simulations

To verify the feasibility and applicability of MDS in the reconstruction of the light source, inverse simulations were performed. The bioluminescence distributions on the surface were simulated with MC at the wavelengths of 610, 630, 650, and 670 nm. The TM method and SD method were used for comparison.

##### (1) Single-Source Case

**Figure 4:**
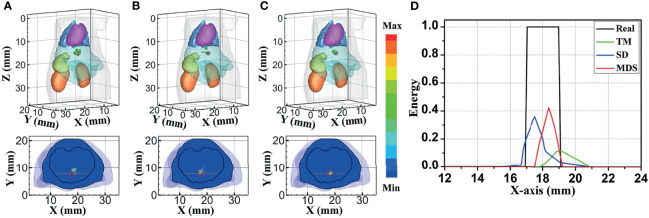
Reconstructed results of the single-source numerical simulations. 3D views of the reconstructed results and corresponding sectional images at Z = 14.8 mm obtained by the **(A)** TM, **(B)** SD, and **(C)** MDS. **(D)** Energy plots along the cut aligned with the x-axis that crosses the sectional images. The cut locations are indicated by the yellow lines in (A–C). TM, traditional multispectral; SD, spectral derivative; MDS, multispectral differential strategy.

**Figure 4** compares the reconstruction performance of the three methods in the single-source reconstruction. The red sphere in the 3D views and the red circle in the sectional images label the actual position of the real source, while the green irregular shapes are the reconstructed sources. It can be found that the TM method is reconstructed with a big deviation from the real source, and the TM method and SD method are reconstructed with poor morphology recovery capability and result in artifacts around the real source. In contrast, the MDS method achieves a better overlap with the real source. **Figure 4D** shows energy plots along the cut aligned with the x-axis that crosses the sectional images. The results show that the position and shape of the source reconstructed by the MDS method are in good agreement with the real source as compared with the other methods. **Table 3** shows the quantitative analysis of these results. The MDS method obtained the lowest condition number, the smallest LE, the best Dice, and CNR among the three approaches. These results indicate that the MDS method performs better in the target location, shape recovery, and image contrast than the other methods in this set of experiments. The time consumption of the source reconstruction by the MDS method is the lowest, and it saved 54.6% and 94.2% of the time as compared with the SD method and TM method, respectively.

**Table 3:** Quantitative results of different methods in single-source reconstruction.

Methods	Reconstructed source center (mm)	Cond (A)	LE (mm)	Dice	CNR	T (s)
TM	(18.05, 8.85, 14.08)	5.26e+11	1.111	0.22	3.09	394.49
SD	(17.75, 8.83, 14.67)	5.85e+11	0.881	0.55	4.84	50.82
**MDS**	**(18.58, 8.11, 14.19)**	**7.97e+9**	**0.855**	**0.56**	**5.74**	**23.07**

LE, location error; CNR, contrast-to-noise ratio; TM, traditional multispectral; SD, spectral derivative; MDS, multispectral differential strategy.

##### (2) Dual-Source Case

**Figure 5:**
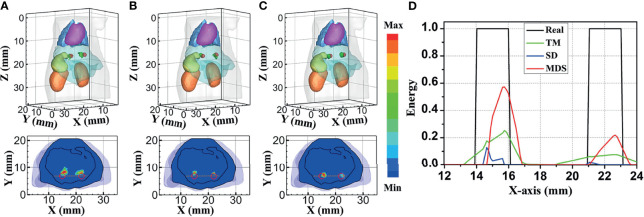
Reconstructed results of the dual-source numerical simulations. 3D views of the reconstructed results and corresponding sectional images at Z = 15.8 mm obtained by the **(A)** TM, **(B)** SD, and **(C)** MDS. **(D)** Energy plots along the cut aligned with the x-axis that crosses the sectional images. The cut locations are indicated by the yellow lines in panels **(A–C)**. TM, traditional multispectral; SD, spectral derivative; MDS, multispectral differential strategy.

To further verify the multi-source resolution performance of the MDS method, dual-source numerical simulation experiments were carried out for the reconstruction. **Figures 5A–C** show the 3D views of the reconstructed results of each method and their sectional images. As the results show, the three methods all accurately located the two light sources, and the MDS method has a lower error in positioning and shape recovery as compared with the TM method and SD method. **Figure 5D** shows energy plots along the cut aligned with the x-axis that crosses the sectional images. The results further verify that the position and shape of the source reconstructed by the MDS method are in good agreement with the real source. The quantitative analysis in **Table 4** confirmed our observation; the MDS method achieved the lowest condition number, the smallest LE, the best Dice, and CNR among the three approaches. The results demonstrate that the MDS method has superior location accuracy, morphology recovery capability, and image contrast capability in dual-source reconstruction. Simultaneously, it has more advantages in time cost in this set of experiments, which saved 23.0% of source reconstruction time as compared with the SD method and reduced 92.0% of that as compared with the TM method.

**Table 4:** Quantitative results of different methods in dual-source reconstruction.

Methods	Reconstructed source center(mm)	Cond (A)	LE (mm)	Dice	CNR	T (s)
TM	(21.80, 8.04, 15.95) (15.73, 8.47, 15.75).	6.79e+11	1.07 1.64	0.37	2.17	726.34
SD	(22.07, 8.05, 15.60) (14.93, 7.74, 16.32)	7.48e+11	1.07 0.91	0.52	3.45	75.41
**MDS**	(22.60, 7.20, 15.84) (15.59, 7.51, 15.70)	3.42e+11	0.63 0.78	0.59	3.79	58.06

LE, location error; CNR, contrast-to-noise ratio; TM, traditional multispectral; SD, spectral derivative; MDS, multispectral differential strategy.

### 3.5 *In Vivo* Experiments

**Figure 6:**
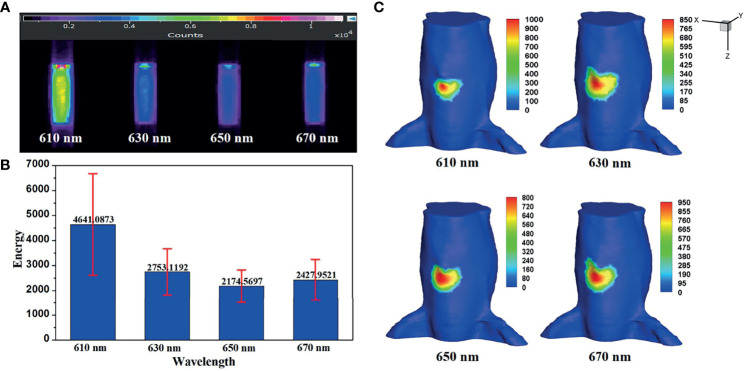
**(A)** The luminescence images of luminescent solution at various wavelengths. **(B)** Their mean value and variance. **(C)** The surface light distributions corresponding to each emission wavelength in *in vivo* experiments.

In a practical BLT system, bioluminescence is a broad spectrum, which can be divided into several wavelength ranges. In Eq. (16) and Eq. (18), for a given wavelength *λn*, the ratio of each spectral energy to the total energy *η_n_* needs to be obtained. **Figure 6A** shows the luminescence images of luminescent solution at various wavelengths captured by the EMCCD with bandpass filters with different wavelengths. The mean value and variance of luminescence energy of each wavelength were calculated, as shown in **Figure 6B**, which were regarded as the energy contribution of the internal light source *S* at each wavelength in the *in vivo* experiments. **Figure 6C** shows the surface light distributions corresponding to each wavelength in *in vivo* experiments.

**Figure 7:**
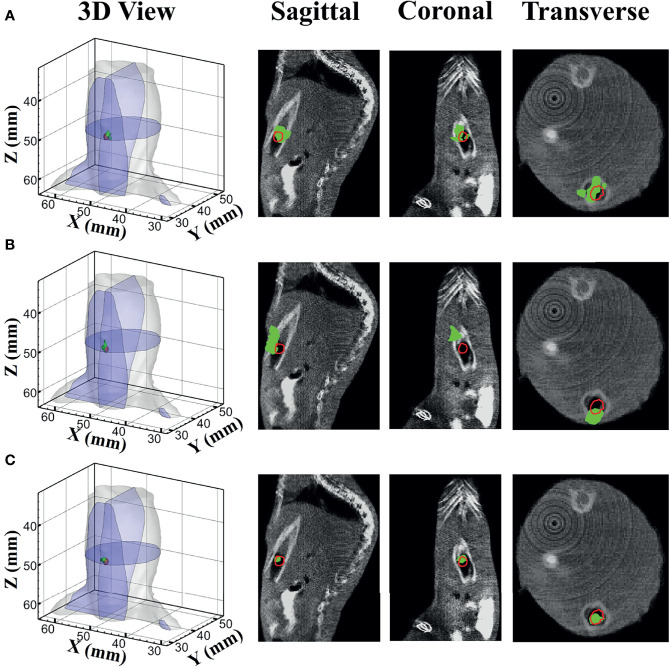
Reconstruction results of *in vivo* experiments. 3D views of the reconstructed results and corresponding sectional images obtained by **(A)** the TM, **(B)** SD, and **(C)** MDS. TM, traditional multispectral; SD, spectral derivative; MDS, multispectral differential strategy.

**Figure 7** shows the reconstructed results of *in vivo* experiments performed with the TM, SD, and MDS methods. The 3D views of the reconstructed results are displayed in the first column, while the real source and reconstructed source positions are represented by red regions and green irregular shapes, respectively. Corresponding sectional images are determined according to the central position of the true source as shown in the next sequence, where the comparison of these with CT results is shown, and the irregularly shaped red circle in the CT sectional images labels the actual position of the real source. The TM method and SD method with poor morphology recovery capability and result in artifacts around the real source and the SD method caused a big deviation from the real source. The MDS method has a lower error in positioning and shape recovery than TM and SD, and the reconstructed source in **Figure 7C** is the closest to the real source in sagittal, coronal, and transverse plane images. The quantitative analysis of the reconstructed source is recorded in **Table 5**. The MDS method achieves the lowest condition number, the smallest LE, the best Dice, and CNR among the three approaches. The results demonstrate that the MDS method performs better in the target location, morphology recovery, and image contrast than the TM method and SD method. In addition, it has more advantages in time cost in this set of experiments, which saved 30.5% of source reconstruction time as compared with the SD method and reduced 78.1% of that as compared with the TM method.

**Table 5:** Quantitative results of *in vivo* experiments.

Methods	Reconstructed source center (mm)	Cond (A)	LE (mm)	Dice	CNR	T (s)
TM	(49.04, 34.42, 48.36)	1.15e+11	0.938	0.36	4.25	228.37
SD	(49.52, 32.96, 47.24)	5.87e+7	1.210	0.17	3.11	71.92
**MDS**	**(50.03, 33.90, 48.06)**	**1.14e+7**	**0.344**	**0.70**	**9.45**	**49.96**

LE, location error; CNR, contrast-to-noise ratio; TM, traditional multispectral; SD, spectral derivative; MDS, multispectral differential strategy.

## 4 Discussion and Conclusions

In previous studies, multispectral reconstruction has attracted remarkable attention since multispectral data can reduce the ill-posedness of BLT and enhance the stability of reconstruction (34). Based on these studies, a new MDS method was proposed for BLT. Rather than directly using bioluminescence images acquired at several wavelengths, the spectral difference between the measured data at similar wavelengths is used to further improve BLT quality. Considering that light at similar wavelengths encounters a near-identical system response, we used the MDS to reduce the errors that resulted from using the DE model and data acquisition of the imaging system.

The effectiveness of the MDS in reducing optical transmission errors was analyzed with the forward simulations. Based on the surface light distributions calculated by MC and DE at the same wavelength, we made a quantitative analysis of energy differences and the cosine similarity between the MC data and the DE data. The changes of the indexes show that using MDS can reduce the errors in the process of light transmission in both the single-source model and dual-source model.

Inverse simulations were performed to verify the feasibility and applicability of the MDS in BLT reconstruction. The bioluminescence distributions on the surface were simulated with MC at wavelengths of 610, 630, 650, and 670 nm as the measured data. Condition numbers of the system matrix constructed by each method were calculated and compared. Compared to the TM method and SD method, MDS had the lowest condition number, which means that the ill-posedness has been partially alleviated. Thus, the MDS method obtained superior location accuracy, morphology recovery capability, and image contrast capability in the source reconstruction. The *in vivo* experiments further verify the performance of the MDS in a practical BLT system. The MDS method achieved the smallest LE, the best Dice, and CNR with the smallest time cost among the three approaches.

It needs to be emphasized that in this work, in the acquisition of *in vivo* multispectral data, the imaging object was kept motionless, so the interference from the change in position of the imaging object on the imaging quality does not exist. In previous studies, a consensus that multispectral data can increase the amount of known boundary measurements and effectively reduce the ill-posedness of the inverse problem has been achieved, although in the acquisition of multispectral data, the influence of time on light signal is universal (34, 42, 43). Moreover, our proposed method can alleviate the noise introduced in the long-time collection of the bioluminescent light signal. In the next study, a multispectral camera with a filter wheel consisting of optical bandpass filters will be used to reduce the acquisition time of multispectral data, and the time-resolved *in vivo* signal will be quantified for having accurate input data for reconstruction.

In conclusion, a new MDS was presented to reduce system errors and improve reconstruction accuracy. Simulations and *in vivo* experiments demonstrated that it performed better in the target location, morphology recovery, and image contrast as compared to the TM method and SD method. This method has a view to provide a more reliable reference for the later research on BLT.

## Data Availability Statement

The original contributions presented in the study are included in the article/supplementary material. Further inquiries can be directed to the corresponding authors.

## Ethics Statement

The animal study was reviewed and approved by the Animal Ethics Committee of the Northwest University of China.

## Author Contributions

YL contributed to the design and implementation of this research and successfully achieved the expected goal. MC made some contributions to the data collection and experiments. HG provided great help on the whole scheme design of this research and the final article. XGH was responsible for the part of data collection and assisted in the animal experiments. XWH and JY provided the research platform with high requirements and rendered some important ideas during our research, and JY provided great help in revising the manuscript. XLH and HY gave guidance on the experiments and manuscripts. All authors listed have made a substantial, direct, and intellectual contribution to the work and approved it for publication.

## Funding

This study was funded by the National Natural Science Foundation of China under Grants 61971350, 61901374, 61906154, and 11871321; the Natural Science Foundation of Shaanxi under Grant 2019JQ-724; Postdoctoral Innovative Talents Support Program under Grant BX20180254; the Scientific and Technological projects of Xi’an under Grant 201805060ZD11CG44; the Key Research and Development Program of Shaanxi 2020SF-036; and Xi’an Science and Technology Project 2019218214GXRC018CG019-GXYD18.3.

## Conflict of Interest

The authors declare that the research was conducted in the absence of any commercial or financial relationships that could be construed as a potential conflict of interest.

## Publisher’s Note

All claims expressed in this article are solely those of the authors and do not necessarily represent those of their affiliated organizations, or those of the publisher, the editors and the reviewers. Any product that may be evaluated in this article, or claim that may be made by its manufacturer, is not guaranteed or endorsed by the publisher.
